# A Case Report of a 5-Year-Old Girl with Self-Limited Epilepsy with Autonomic Seizures

**DOI:** 10.3390/pediatric15030045

**Published:** 2023-08-11

**Authors:** Georgios Katsaras, Petrina Samartzi, Pelagia Tsitsani

**Affiliations:** Paediatric Department, General Hospital of Pella—Hospital Unit of Edessa, 58200 Edessa, Greece; petrinasamartzi@gmail.com (P.S.); pelazina@gmail.com (P.T.)

**Keywords:** Panayiotopoulos syndrome, SeLEAS, childhood, seizure, epilepsy

## Abstract

Background: Self-limited epilepsy with autonomic seizures (SeLEAS), formerly known as Panayiotopoulos syndrome (PS), is a common multifocal autonomic childhood epileptic syndrome. SeLEAS affects 6% of children in between the ages of 1 and 15 years who have had one or more afebrile seizures in their lifetime. Case: A 5-year-old girl was admitted to the paediatric emergency room (ER) of our hospital due to a reported episode of vomiting during her sleep, followed by central cyanosis perorally of sort duration (<5′), a right turn of her head, and gaze fixation with right eye deviation. She was dismissed after a one-day hospitalization free of symptoms. A month later, the patient was admitted to the paediatric ER of a tertiary health unit due to a similar episode. The patient underwent EEG, which revealed pathologic paroxysmal abnormalities of high-amplitude sharp waves and spike-wave complexes in temporal-occipital areas of the left hemisphere, followed by enhancement of focal abnormalities in temporal-occipital areas of the left hemisphere during sleep. The patient was diagnosed with SeLEAS and started levetiracetam. Conclusions: SeLEAS can be easily misdiagnosed as many physicians may not be very familiar with this disease, and, on the other hand, the autonomic manifestations can be easily disregarded as seizures. The physician must always be alert and search beneath the symptoms to find the cause rather than only treat them.

## 1. Introduction

Self-limited epilepsy with autonomic seizures (SeLEAS), previously known as Panayiotopoulos syndrome (PS) or early-onset benign occipital epilepsy, is a well-known childhood epileptic syndrome characterized by multifocal autonomic seizures. Renowned neurologist Chrysostomos Panayiotopoulos was instrumental in describing this syndrome [1]. It is characterized by seizures, often prolonged, with predominantly autonomic symptoms (e.g., nausea and vomiting, urinary and faeces incontinence, mydriasis or mysis, arrhythmias, blood pressure alterations, and pallor/cyanosis or flushing), and by specific electroencephalogram (EEG) showing shifting and/or multiple foci, often with occipital predominance [2,3]. Rarely visual symptoms, such as hallucinations or loss of vision, may be presented during the seizures [4]. According to the new International League against Epilepsy (ILAE) classification, former PS is currently described as SeLEAS [5].

SeLEAS affects 6% of children in between the ages of 1 and 15 years who have had one or more afebrile seizures in their lifetime. The percentage seems to be increased by up to 13% for those in the 3–6-year-old age group, with the peak age at 4–5 years. This condition affects boys and girls equally, with an estimated prevalence of 2–3 cases per 1000 children in the general population [6,7].

One of the challenges in diagnosing SeLEAS is its potential to mimic various epileptic and non-epileptic disorders, making a comprehensive differential diagnosis crucial. Conditions such as atypical migraine, gastroenteritis, motion sickness, syncope, sleep disorders, intoxication, focal or diffuse brain lesions, cyclic vomiting syndrome, self-limited epilepsy with centrotemporal spikes (SeLECTS, formerly known as Rolandic epilepsy), febrile seizures, and acute encephalopathy should be considered [8]. In 90% of SeLEAS cases, the EEG reveals multifocal functional spikes that are amplified by sleep. In SeLEAS, functional spikes appear in many brain locations, often shifting from one area to another area in the EEG series [2].

Despite this fact, that SeLEAS is a well-described syndrome, its diagnosis is often missed. This is why the primary objective of our study was to present a detailed case report of a preschool girl diagnosed with SeLEAS, providing comprehensive insights into her clinical presentation and management. Furthermore, we meticulously reviewed the most recent literature on SeLEAS to provide a comprehensive overview of the current understanding of its clinical features, diagnostic challenges, and management approaches. Through this study, we sought to enhance awareness and improve the management of patients with SeLEAS, ultimately leading to better outcomes and quality of life for individuals affected by this syndrome.

## 2. Case Report

A 5-year-old girl was admitted to the paediatric emergency room (ER) of our hospital due to a reported episode of vomiting during her sleep, followed by central cyanosis perorally of short duration (<5′). She also experienced a sudden rightward turn of her head and gaze fixation with right eye deviation. Additionally, urinary and faecal incontinence were observed. After the episode, the patient appeared drowsy. It was noted that she had a similar episode approximately one year prior.

Our patient was admitted to the paediatric department for further investigation. Laboratory screening (haematological and general biochemical examinations), electrocardiogram (ECG) monitoring, and ophthalmological assessment with fundoscopy took place.

Considering the patient’s medical history, the suspicion of SeLEAS arose as a potential diagnosis for her episodes. After a one-day hospitalization without any recurrence of symptoms, the patient was discharged, with the recommendation of neurological assessment by a paediatric neurologist and EEG evaluation.

About a month later, the patient experienced a similar episode and was subsequently readmitted to the paediatric ER, this time at a tertiary health unit. Further investigations, including an EEG, were carried out.

**Figure 1:**
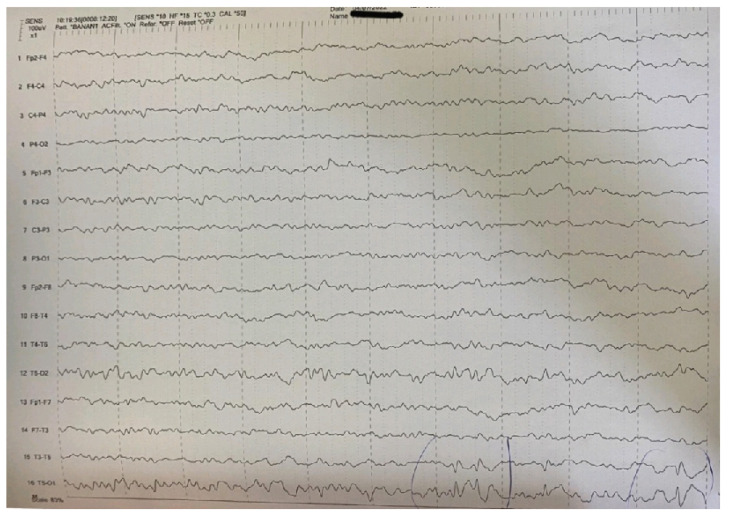
High-amplitude sharp waves and spike-wave complexes in temporal-occipital areas of the left hemisphere in the EEG.

**Figure 2:**
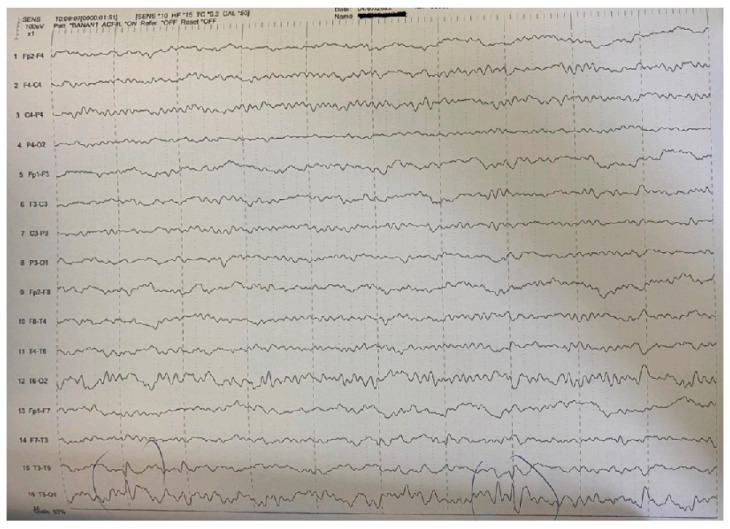
Enhancement of focal abnormalities in temporal-occipital areas of the left hemisphere in the EEG during sleep.

The EEG revealed pathologic paroxysmal abnormalities of high-amplitude sharp waves and spike-wave complexes in temporal-occipital areas of the left hemisphere (Figure 1), followed by enhancement of focal abnormalities in temporal-occipital areas of the left hemisphere during sleep (Figure 2).

Based on the EEG findings, our first hypothesis was confirmed, and the patient was diagnosed with SeLEAS and started levetiracetam as prophylactic therapy.

## 3. Discussion

SeLEAS is probably the early-onset phenotype of a maturation-related childhood seizure-susceptibility syndrome [2]. Epileptic discharges generated at various cortical locations that influence vulnerability for children in terms of emetic centres and the hypothalamus, the principal regulatory centre of the autonomic nervous system, have been proposed to be the cause of emesis and the involvement of the autonomic system [7]. These symptoms in SeLEAS are specific to childhood, and they do not occur in adults. The fundamental manifestations of SeLEAS consist of autonomic epileptic seizures and autonomic status epilepticus. The initial seizure semiology is usually vomiting (emesis); however, other autonomic manifestations such as nausea, retching, hypersalivation, urinary and/or faeces incontinence, mydriasis or mysis, cardiac arrhythmias, blood pressure changes, and pallor/cyanosis or flushing are also common [3]. Two-thirds of the seizures start in sleep. The child displays either similar complaints upon waking or exhibits symptoms such as vomiting, consciousness issues, confusion, or unresponsiveness [2]. In Table 1, we present the symptoms and manifestations of our patient in contrast to those described in the literature.

Regarding SeLEAS, 30% of the seizures are relatively brief and self-limited within 2 to 10 min. The remaining 70% of the seizures are long-lasting (more than 10 min) or status epilepticus (more than 30 min to hours) [9]. The child, who is the subject, can experience seizures that vary in duration, ranging from short to extended periods. These seizures may exhibit noticeable or subtle autonomic manifestations. Even after the most severe and lengthy seizures, the patient is normal after a few hours of sleep [2]. In childhood, SeLEAS is the primary underlying factor for afebrile nonconvulsive status epilepticus, which is the most prevalent cause [5].

Ten to twenty percent of autonomic seizures and autonomic status epilepticus in children, similar and with the same sequence as in SeLEAS, may be the result of cerebral pathology of any type [10,11] or Angelman’s syndrome [12,13]. These diagnoses are easy to make based on abnormal neurological or mental signs, abnormal brain imaging, and EEG background abnormalities [10,11]. Seizures are infrequent in most patients with SeLEAS, with 25% only having a single seizure and the majority having fewer than five seizures in total. The epilepsy is considered self-limited, with remission typically within a few years from onset. The mean duration of the syndrome is approximately 3 years. Nevertheless, MRI should be considered in cases with recurrent seizures or atypical presentations [5] and some patients should be monitored for possible neurobehavioural comorbidities [14].

SeLEAS is often misdiagnosed [7,9,15,16,17]. The main reason for this is that emetic and other autonomic manifestations are not recognized as seizure events [2]. In order to exclude drug exposure or substance abuse, toxicology screening should be considered. If meningitis or encephalitis is suspected, lumbar puncture is the examination that will confirm the diagnosis. EEG helps in determination of seizure type, epilepsy syndrome, and risk for recurrence and, therefore, may affect further management decisions [9]. Although, SeLECTS and SeLEAS are self-limited and management is similar, they are clinically different. Emesis has not been reported in SeLECTS. Furthermore, seizures in SeLECTS are usually brief compared to characteristic seizures in SeLEAS [7]. Febrile seizures can be identified when seizures occur either inadvertently or due to changes in ictal thermoregulation that coincide with a fever. However, this has no adverse prognostic implications, because febrile seizures are also benign and age-related [2]. As for the clinical manifestation of migraine including flickering achromatic or black and white, linear and zigzag patterns in the centre of the visual field, a 4–30 min time duration, and scotoma, they do not appear in SeLEAS. Conversely, eye deviation and other typical convulsive manifestations of SeLEAS do not manifest in these migraines [7].

It is well-established knowledge that genetic factors are associated with idiopathic focal epilepsy syndromes [18,19]. The SCN1A gene, that codes for the a1 subunit of the neuronal sodium channel, is considered to be the most clinically relevant epilepsy gene [20,21]. Many different mutations in the gene have been described regarding the familial epilepsy syndrome of generalized epilepsy with febrile seizures plus (GEFS+). The most clinically significant mutations in the SCN1A gene are associated with Dravet syndrome [22], while mutations in this gene have been associated as well with severe myoclonic epilepsy in infancy (Dravet syndrome), familial hemiplegic migraine, and developmental and epileptic encephalopathy (Table 2) [23]. Nevertheless, mutations in this gene have also been detected in several confirmed cases of SeLEAS [22,24,25]. These findings suggest that the GEFS+ spectrum could extend to self-limited focal epilepsies in childhood, such as SeLEAS [24].

Most appropriate management of SeLEAS includes education about the nature and prognosis of the disease. Antiepileptic medication as prophylactic treatment may not be needed for most of the patients with SeLEAS. However, 10% to 20% of patients may present with recurring seizures or have autonomic status epilepticus for several days and may benefit from antiepileptic medication. In addition, most parents usually request treatment because of the dramatic psychosocial impact of seizures on themselves [26,27,28]. Of the established antiseizure medication, carbamazepine and sodium valproate appear to be equally appropriate [29]. Sodium valproate should only be used in women of childbearing potential when all other treatments are ineffective or not tolerated [30]. Carbamazepine has been reported to lead to seizure exacerbation in some cases [31]. Levetiracetam has also been used with excellence response in seizure control and fewer side effects [29,32,33].

Autonomic status epilepticus (Aut SE) is considered to be a critical medical emergency in paediatric cases. Recognizing the urgency of Aut SE and promptly initiating appropriate treatment is essential to prevent further complications and ensure the well-being of the affected child [9,34]. In the context of SeLEAS, Aut SE typically presents with specific symptoms in affected children. If the child is awake during an episode, they may initially complain of feeling unwell. This is often followed by episodes of retching and vomiting, accompanied by noticeable pallor, alterations in cardiac rhythm, mydriasis (dilated pupils), and disruptions in thermoregulation. As the episode progresses, the child’s level of awareness and responsiveness become impaired, frequently accompanied by eye and/or head deviation. Subsequently, a prolonged phase of fluctuating consciousness may ensue, ultimately culminating in a brief convulsive seizure [3]. To promptly terminate the nonconvulsive status epilepticus, the appropriate treatment involves the administration of benzodiazepines. These can be delivered intravenously or through rectal or buccal preparations [2,29]. It is crucial to exercise caution when administering aggressive treatment, as it carries the risk of iatrogenic complications, including cardiorespiratory arrest. Therefore, a balanced approach is necessary to mitigate potential harm. Early intervention, typically initiated by parents or caregivers, using rectal or buccal benzodiazepines has been shown to be more effective than delayed emergency treatment [9,34]. Further research and advancements in the management of Aut SE are warranted to optimize treatment strategies and minimize potential risks associated with its management.

## 4. Conclusions

Self-limited epilepsy with autonomic seizures SeLEAS is characterized by the occurrence of focal autonomic seizures, typically of prolonged duration, during early childhood. However, SeLEAS can often be misdiagnosed due to the prominence of gastrointestinal symptoms and other autonomic manifestations, which may erroneously be attributed to non-epileptic origins.

To avoid misdiagnosis, physicians must adopt a comprehensive approach that involves a detailed medical history, thorough physical examination, and specialized diagnostic tests. The medical history should specifically inquire about the characteristic features of autonomic seizures, including their onset, duration, and associated symptoms. A careful assessment of the clinical presentation, coupled with the interpretation of electroencephalography (EEG) findings, can provide valuable insights into the nature of the seizures and aid in confirming the diagnosis of SeLEAS.

## Figures and Tables

**Table 1:** Symptoms and manifestations of SeLEAS [3,4] in comparison to our patient.

Symptoms and Manifestations of SeLEAS	Characteristics and Frequency of Symptoms and Manifestations of SeLEAS	Symptoms and Manifestations of Our Patient ^1^
Age of onset (years)	1–14 (Mean: 4–5)	5 years old
Duration of seizures		
Less than 4 min	Rare	<5 min duration of seizures
≥5 min	As a rule	
Seizures during sleep	>2/3 of cases	Seizures during sleep
Features of seizures		
Ictal vomiting	Frequent	Ictal vomiting
Deviation of the eyes	Frequent	Deviation of the eyes
Impairment of consciousness	Frequent	Drowsiness
Visual hallucinations	Rare	
Loss of vision	Rare	
Autonomic semiology	As a rule	Urinary and faecal incontinence
Postictal migraine-like headache	Rare	
Seizures evolving into autonomic status epilepticus	Frequent	
Main interictal EEG	Multifocal spikes	High-amplitude sharp waves and spike-wave complexes in temporal-occipital areas of the left hemisphere in the EEG. Enhancement of focal abnormalities in temporal-occipital areas of the left hemisphere in the EEG during sleep.

EEG: electroencephalogram; SeLEAS: self-limited epilepsy with autonomic seizures. ^1^ As reported by the patient’s parents.

**Table 2:** Clinical epilepsy syndromes and less common clinical presentations reported in individuals with SCN1A seizure disorders.

Clinical Epilepsy Syndromes	Less Common Presentations
Febrile seizures (simple or complex)	Epilepsy with focal seizures
Febrile seizures plus (FS+)	Myoclonic–astatic epilepsy (MAE, Doose syndrome)
Generalized epilepsy	Lennox–Gastaut syndrome
Generalized epilepsy with febrile seizures plus (GEFS+)	Infantile spasms
Dravet syndrome	Vaccine-related encephalopathy and seizures
Severe myoclonic epilepsy, borderline (SMEB)	
Intractable childhood epilepsy with generalized tonic–clonic seizures (ICE-GTC)	
Infantile partial seizures with variable foci	

Miller and Sotero de Menezes. SCN1A Seizure Disorders; 1993 [23].

## Data Availability

Not applicable.
